# The microbiome composition of *Aedes aegypti* is not critical for *Wolbachia*-mediated inhibition of dengue virus

**DOI:** 10.1371/journal.pntd.0005426

**Published:** 2017-03-07

**Authors:** Michelle D. Audsley, Yixin H. Ye, Elizabeth A. McGraw

**Affiliations:** School of Biological Sciences, Monash University, Clayton VIC, Melbourne, Australia; The Pennsylvania State University, UNITED STATES

## Abstract

**Background:**

Dengue virus (DENV) is primarily vectored by the mosquito *Aedes aegypti*, and is estimated to cause 390 million human infections annually. A novel method for DENV control involves stable transinfection of *Ae*. *aegypti* with the common insect endosymbiont *Wolbachia*, which mediates an antiviral effect. However, the mechanism by which *Wolbachia* reduces the susceptibility of *Ae*. *aegypti* to DENV is not fully understood. In this study we assessed the potential of resident microbiota, which can play important roles in insect physiology and immune responses, to affect *Wolbachia*-mediated DENV blocking.

**Methodology/Findings:**

The microbiome of *Ae*. *aegypti* stably infected with *Wolbachia* strain *w*Mel was compared to that of *Ae*. *aegypti* without *Wolbachia*, using 16s rDNA profiling. Our results indicate that although *Wolbachia* affected the relative abundance of several genera, the microbiome of both the *Wolbachia-*infected and uninfected mosquitoes was dominated by *Elizabethkingia* and unclassified *Enterobacteriaceae*. To assess the potential of the resident microbiota to affect the *Wolbachia-*mediated antiviral effect, we used antibiotic treatment before infection with DENV by blood-meal. In spite of a significant shift in the microbiome composition in response to the antibiotics, we detected no effect of antibiotic treatment on DENV infection rates, or on the DENV load of infected mosquitoes.

**Conclusions/Significance:**

Our findings indicate that stable infection with *Wolbachia* strain *w*Mel produces few effects on the microbiome of laboratory-reared *Ae*. *aegypti*. Moreover, our findings suggest that the microbiome can be significantly altered without affecting the fundamental DENV blocking phenotype in these mosquitoes. Since *Ae*. *aegypti* are likely to encounter diverse microbiota in the field, this is a particularly important result in the context of using *Wolbachia* as a method for DENV control.

## Author summary

Dengue virus is transmitted by the mosquito *Aedes aegypti* and can cause dengue fever and dengue haemorrhagic fever in humans. The World Health Organization currently considers it as the most important mosquito-borne virus globally. One method to control dengue infection of *Ae*. *aegypti* is to infect the mosquito with a common bacterium, *Wolbachia*, which increases the mosquito’s resistance to dengue virus. The mechanism by which resistance to dengue virus occurs is not well understood. Here, we considered whether other bacteria that reside in the mosquito might affect the ability of *Wolbachia* to limit dengue virus infection. *First*, we assessed whether *Wolbachia* had an impact on the abundance of bacterial species present in *Ae*. *aegypti*, finding that it had minimal effects. *Second*, we altered the composition of the bacterial species present by treating *Ae*. *aegypti* with antibiotics, then examined whether this affected *Wolbachia’s* antiviral effect. We found that there was no difference in the susceptibility of the mosquitoes to dengue virus, regardless of antibiotic treatment. We therefore conclude that it is unlikely that there are specific resident bacteria required for the principal mechanism(s) by which *Wolbachia* reduces susceptibility of *Ae*. *aegypti* to dengue virus.

## Introduction

Dengue virus (DENV) is an RNA arbovirus and the causative agent of dengue fever and the more severe dengue haemorrhagic fever. There are four serotypes of DENV (DENV1-4), which together are estimated to infect 390 million people per year [1]. Currently there are no specific antiviral therapies approved to treat DENV infection, and new DENV vaccines do not provide optimal protection (World Health Organization, 2016). The primary vector of DENV is the mosquito *Aedes aegypti* whose global range is expanding in part due in to urbanisation and climate change [2,3]. Strategies for DENV control *via* the vector have traditionally relied on insecticide application, but recent approaches using genetic modification and a symbiotic bacterium of insects called *Wolbachia* are being tested in field trials [4–7].

*Wolbachia* is an obligately intracellular bacterium transmitted from females to their offspring, and a common member of the resident microbiota in insects [8]. Although not naturally found in *Ae*. *aegypti*, stably inherited infections of *Wolbachia* have been created by transinfection [9,10]. In naturally infected insects, *Wolbachia* has been shown to limit virus replication [11,12]. In *Ae*. *aegypti* this effect extends to important human pathogens including DENV, Zika virus, yellow fever virus and Chikungunya virus [10,13–15]. While the mechanism of *Wolbachia’s* antiviral effect is poorly understood, there is some evidence for the contribution of nutritional competition, priming (pre-activation) of the mosquito immune response, and altered host miRNA toward the phenotype [16–18]. In addition to the complex interactions between mosquito host and *Wolbachia* there is also the potential that additional bacterial players in the microbiome could mediate mosquito susceptibility to viruses [19,20].

One key mechanism by which residents of the microbiome can mediate viral susceptibility is through changes in the expression of insect immunity. In *Drosophila*, for instance, the presence of specific gut bacterium is necessary to fully activate an antiviral response that viral infection alone does not trigger [21]. In *Ae*. *aegypti*, removal of the gut microflora by antibiotic treatment reduced expression of key immune genes and reduced DENV titres in the midgut [22]. A related study also found that re-introduction of the bacteria *Proteus* and *Paenibacillus* to the *Ae*. *aegypti* midgut after antibiotic treatment caused significant reduction in DENV titre, with data indicating that the re-introduction of these bacterial genera upregulated immune effector gene expression to cause an antiviral effect [23]. Together these studies suggest that the presence or absence of specific bacterial taxa can alter the activation of insect immune responses and consequently affect the capacity for viral infection.

The microbiome could also have indirect effects on mosquito susceptibility to viruses either *via Wolbachia* or the host. For instance, bacteria such as *Asaia* and *Spiroplasma* have been found to have negative effects on *Wolbachia* transmission and/or density [24–26]. *Wolbachia* and *Spiroplasma* have been shown to interact in *Drosophila* in a manner by which *Wolbachia* density is reduced by *Spiroplasma* co-infection, but *Spiroplasma* is unaffected by *Wolbachia* [24]. In the mosquito, a reduction in *Asaia* abundance following antibiotic treatment improved vertical transmission of *Wolbachia* by *Anopheles gambiae* and also reduced mortality induced by blood-meal in *Anopheles* *stephensi* [25]. These studies suggest that *Wolbachia* may have specific interactions with the native microbiome that could affect vector competence. More broadly, resident microbiota are known to play key roles in mosquito biological functions/fitness. For example, bacteria-free mosquito larvae do not develop past the first instar, but colonization with one of several strains of bacteria can rescue development [27]. Changes in mosquito fecundity have also been reported following antibiotic treatment [28,29]. Since the maintenance and induction of immunity is costly for the host [30], it is possible that broad effects of the microbiome on insect fitness or condition may shift the balance in the potential trade-off between immunity and fitness, thereby indirectly impacting on susceptibility to viruses.

Given these findings, it is possible that members of the mosquito microbiota are playing either direct or indirect roles in *Wolbachia*-mediated pathogen blocking. Assessing the involvement of these ‘third-parties’ is critical as it may affect the efficacy of *Wolbachia’s* blocking effects across populations in the field. A recent study that profiled the microbiome of *Ae*. *aegypti* demonstrated its composition varied heavily even over short geographic distances [31]. Here we have deliberately manipulated the microbiome of *Ae*. *aegypti* and assessed whether there are *Wolbachia*-by-microbiome interactions that may affect *Wolbachia*-mediated blocking. The *Wolbachia* and mosquito strains examined hail from the original field release trial populations in Australia and hence the findings may be relevant to other sites globally where the same *Wolbachia* strain is currently being released for DENV control [7].

## Methods

### Ethics statement

The DENV strain ET300 used in this study was obtained from researchers associated with both Queensland Health (Australia) and the University of Queensland. IRB approval was obtained from the latter. Patient data were anonymised by the former. Human volunteer blood-feeding of mosquitoes was approved by the Monash University Human Research Ethics Committee (ethics number CF11/0766–2011000387), and the participant provided written informed consent.

### Mosquito rearing

The wildtype (wt) *Ae*. *aegypti* line was propagated from mosquitoes collected from Babinda, Australia and used within 5 generations in the laboratory. Mosquito collection from private land was performed with permission from the owners/residents. The *Wolbachia*-infected *w*Mel *Ae*. *aegypti* line has been described previously [10] and was generation F_22_; 10% wt males were introduced into the *w*Mel line at each generation to maintain a uniform genetic background and retain the *Wolbachia* infection status [32]. Adult mosquitoes were reared at 26°C and 65% humidity with a 12 h light/dark cycle. All larvae were maintained with fish food pellets (Tetramin, Tetra). Adult mosquitoes were fed with 10% sucrose solution, without or with penicillin-streptomycin (10 U / ml, 10 μg / ml) and kanamycin (200 μg / ml) as in [25], for 3 successive generations. Females were blood-fed by a single human volunteer for the first two generations, before the third generation was used in DENV-infection experiments.

### Virus strains and infection of mosquitoes

DENV strain ET300 was propagated in C6/36 mosquito cell line by infection at MOI of 0.01 in RPMI medium supplemented with 2% Fetal Bovine Serum (FBS), L-glutamine and 1 M HEPES buffer. Seven days post-infection (DPI) supernatant was harvested and clarified by centrifugation at 12,000 ×g at 4°C. Virus was used immediately for inoculation of defibrinated sheep blood at a 1:1 ratio, and DENV titre was determined retrospectively by plaque assay as a final concentration of 1 x 10^6^ plaque forming units (PFU) / ml. Mosquitoes were starved for 24 to 30 h before provision of the DENV-blood-meal for two to three hours through a piece of porcine intestine, using artificial feeders heated to 37°C. The following day mosquitoes were sorted based on feeding, with unfed mosquitoes discarded.

### Sample collection

Females were collected for processing at seven to eight days post-feed. Each mosquito was surface sterilized using 10% bleach, followed by a wash in 80% ethanol and a rinse in sterile water. To reduce bias in 16s rDNA profiling due to high levels of *Wolbachia* sequence in the *w*Mel line, ovaries (the major reservoir for *Wolbachia* [10]) were removed from bodies of all treatment groups. Heads were also removed for intended use as a proxy to detect DENV dissemination but DENV genome copy numbers in wt *Ae*. *aegypti* heads did not consistently have high enough detectable titres. Thus, all analysis of DENV genome copies and 16s profiling were performed on the *Ae*. *aegypti* bodies. Dissections were performed in sterile 1 × phosphate buffered saline (PBS) using sterilized needle/forceps. Individual bodies were stored in sterile 0.1 ml 1 × PBS, homogenised using sterile 3 mm glass beads in a mechanical homogenizer, and stored at– 80°C until processing for genomic DNA (gDNA) extraction and DENV detection.

### Quantitative DENV RT-PCR analysis

For detection of DENV genome copies, one tenth of the volume of the homogenised sample was removed and added to an extraction buffer solution (10 mM Tris pH 8.2, 1 mM EDTA, 50 mM NaCl and proteinase K [33]) in a 1:1 ratio. Samples in extraction solution were incubated in a thermal cycler at 56°C for 5 min followed by 95°C for 5min [33] and then cooled on ice until use. DENV genome copies were determined by a 1-step quantitative reverse-transcriptase PCR (qRT-PCR) using TaqMan Fast Virus 1-Step Master Mix (ThermoFisher Scientific). Reactions contained 2.5 μl of sample, 4 × master mix, 250 μM forward primer, 250 μM reverse primer and 250 μM TaqMan FAM hydrolysis probe in a total 10 μl reaction volume. TaqMan primers and probes complementary to the 3’ untranslated region of DENV and the creation of the DENV-2 standard curve are described elsewhere [17,34]. Thermocycling conditions were as recommended by the manufacturer. Percentages of individuals infected with DENV were calculated; the lower detection limit for DENV-positive individuals was determined by detection of standards and set at 100 copies per qRT-PCR reaction.

### Microbiome profiling

gDNA was extracted from individual mosquito bodies using ReliaPrep gDNA Tissue Miniprep system (Promega) according to the manufacturer’s instructions. gDNA was extracted from each mosquito individually, with final concentrations of 10–35 ng / μl used for bacterial 16s rDNA profiling by Illumina MiSeq (Australian Centre for Ecogenomics, University of Queensland). Samples were amplified (25 cycles) using Q5 HotStart 2X Master Mix (New England BioLabs) with a primer pair for the V3 and V4 regions of bacterial small subunit (SSU) ribosomal gene (16s) (Australian Centre for Ecogenomics primer pair Bac_SSU_341F-806wR: 341F CCTACGGGNGGCWGCAG; 806R GACTACHVGGGTATCTAATCC). For each sample, 2.5 μl of gDNA was used in a total reaction volume of 25 μl. Cycling conditions were as per manufacturer’s specifications with the exception of the initial denaturation that was performed for 2 minutes; annealing temperature was 55°C.

### Bioinformatic and statistical analysis

Initial bioinformatic processing of 16s raw sequence data was performed by the Australian Centre for Ecogenomics (University of Queensland). In brief, sequences were trimmed to remove primers and poor quality sequence, and then hard trimmed to 250 bases (or excluded where less than 250 bases). Two samples with less than 5,000 reads post-filtering were excluded from further analysis. QIIME was used to process files using pick_open_reference_otus.py workflow. Operational taxonomic units (OTU) were BLASTed against the Greengenes database (v 2015/05). Relative abundance of OTU was calculated using BIOM.

Further bioinformatics processing was performed to remove OTU that represented less than 0.1% of sequence reads within a sample; alternative analysis using a cut-off of 10 counts in the raw sequence reads yielded similar results. Data were then grouped by genus and normalised such that the OTU were expressed as a fraction of 1. Statistical analysis was performed on relative abundance at the genus level using SPSS software (SPSS statistics version 22, SPSS Inc, an IBM Company). The effects of *Wolbachia* and antibiotic treatment on genera were assessed using a multivariate general linearized model, with p values of < 0.05 considered significant. DENV copy numbers were log transformed and the effect of *Wolbachia* infection and antibiotic treatment were tested using a one-way ANOVA.

## Results

### Treatment of *Ae*. *aegypti* with penicillin-streptomycin-kanamycin alters the microbiome composition without affecting relative abundance of *Wolbachia*

To examine the role of the native microbiome in *w*Mel-mediated inhibition of DENV in *Ae*. *aegypti* we experimentally manipulated the microbiome of wt (no *Wolbachia* infection) and *w*Mel (stably infected with *Wolbachia* strain *w*Mel) *Ae*. *aegypti* lines before comparing their susceptibility to DENV infection. To alter the microbiome composition, wt and *w*Mel *Ae*. *aegypti* lines were treated with a penicillin-streptomycin-kanamycin combination for three generations before DENV-infection by blood-meal. Rearing, DENV infection and processing were performed in parallel with untreated mosquitoes. The microbiome compositions were compared at seven to eight days post-blood-meal for 19 or 20 individual mosquitoes per treatment group using 16s rDNA profiling by Illumina sequencing.

**Fig 1:**
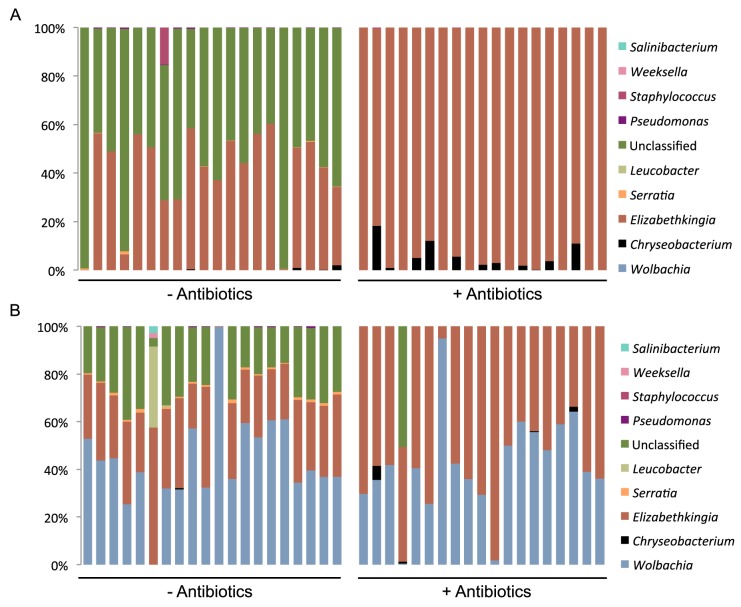
Microbiome composition of wt and *w*Mel *Ae*. *aegypti* mosquitoes. Relative abundance of microbiota present in wt (A) or *w*Mel (B) *Ae*. *aegypti* lines as determined by sequencing of 16s rDNA following a DENV-infectious blood-meal. Where indicated (+ antibiotics) mosquitoes were treated with a combination of penicillin-streptomycin-kanamycin for 3 generations before profiling. Each bar represents a single mosquito. OTU are grouped by genus; ‘unclassified’ indicates OTU that were not classified at the genus level.

There were five taxa that were above our lower limit threshold (OTU representing less than 0.1% of sequences per mosquito) and could be classified at the genus taxonomic level in the wt line (Fig 1A, S1 Table), and eight taxa in the *w*Mel line (Fig 1B, S1 Table). In both *Ae*. *aegypti* lines there were also OTU that made up a substantial proportion of the profile that were unable to be classified at genus level (represented as ‘unclassified’, Fig 1). All OTUs in the unclassified category for wt mosquitoes belonged to the family *Enterobacteriaceae*, whereas for the *w*Mel mosquitoes this comprised both *Enterobacteriaceae* and additional OTUs whose lowest taxonomic classification was the order *Chromatiales*. *Elizabethkingia* (family *Flavobacteriaceae*) and unclassified taxa were the dominant taxa in the wt line, accounting for on average 40% and 58% of OTUs, respectively. In the *wMel* line the dominant taxon identified was *Wolbachia* (average 44%, Fig 1B), but *Elizabethkingia* and unclassified taxa were also present at a high relative abundance, representing on average 29% and 24% of OTUs, respectively.

To assess the success of our antibiotic treatment regime in altering the microbiome profile of both lines we performed statistical analysis by ANOVA, which indicated a significant shift in antibiotic-treated wt (F = 56.168, p < 0.0005, df = 32) and *w*Mel (F = 19.817, p < 0.0005, df = 30) mosquitoes. In both wt (Fig 1A) and *w*Mel (Fig 1B) lines, the taxa with the largest reduction in relative abundance following antibiotic treatment were those unclassified at the genus level (wt: F = 189.105, p < 0.0005, df = 1; *w*Mel: F = 36.679, p < 0.0005, df = 1). *Elizabethkingia* largely replaced those taxa that were reduced in relative abundance due to antibiotic treatment, increasing in relative abundance to represent on average 97% of wt OTUs (F = 158.771, p < 0.0005, df = 1) and 55% of *w*Mel OTUs (F = 28.051, p < 0.0005, df = 1). Antibiotic treatment also caused a rise in *Chryseobacterium* in both lines, but this was only statistically significant in the wt mosquitoes (wt: F = 7.755, p = 0.008, df = 1; *w*Mel: F = 2.061, p = 0.16, df = 1). As expected [25,35], our antibiotic treatment regime caused no changes in the abundance of *Wolbachia* relative to the overall microbiome (F = 0.075, p = 0.785, df = 1), nor relative to a *Ae*. *aegypti* housekeeping gene (S1 Fig). Thus, our antibiotic treatment regime successfully and measurably manipulated the microbiome composition of both lines, without affecting *Wolbachia* abundance.

### Effect of *Wolbachia* on the microbiome profile of non-treated and antibiotic-treated *Ae*. *aegypti*

**Fig 2:**
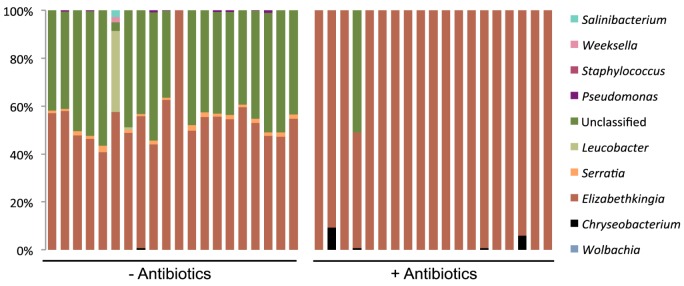
Relative abundance of bacterial genera corrected for *Wolbachia*. Relative abundance of genera from 19 (- antibiotics) or 20 (+ antibiotics) *w*Mel mosquitoes after OTU assigned to *Wolbachia* were removed; ‘unclassified’ indicates OTU that were not classified at the genus level.

Due to its dominance in the community, the presence of *Wolbachia* clearly affected the relative abundance of other genera in the microbiome profile. Thus, to assess the effect of *Wolbachia* on the abundance of other genera relative to each other, we also calculated a “*Wolbachia-*corrected” 16s profile by removing all reads assigned to *Wolbachia* and normalising the remaining OTUs as a fraction of 1 (Fig 2 and S2 Table). When *Wolbachia* was excluded from the profile, the dominant taxa in the *w*Mel line were *Elizabethkingia* (mean *Wolbachia-*corrected relative abundance 55%) and the unclassified taxa (mean *Wolbachia-*corrected relative abundance 42%), similarly to the wt line (compare Fig 1A and Fig 2).

**Fig 3:**
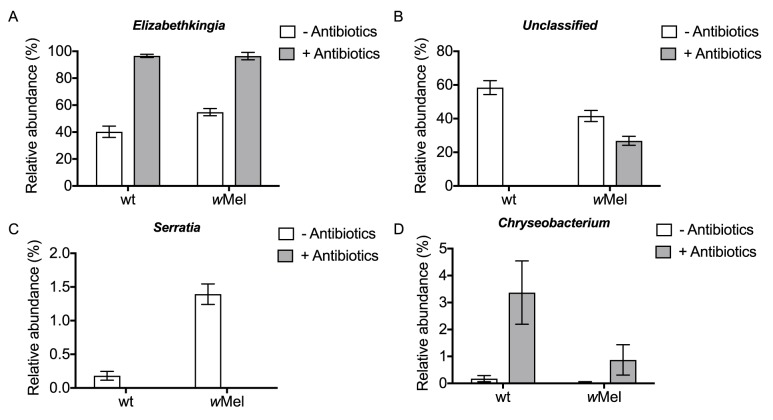
Effects of *Wolbachia* and antibiotic treatment on relative abundance of taxa. Mean relative abundance of *Elizabethkingia* (A), unclassified taxa (B), *Serratia* (C) and *Chryseobacterium* (D) calculated from the *Wolbachia-*corrected microbiome profiles based on 16s sequencing data (S2 Table) and expressed as a percentage of the total.

Between-subjects effects analysis (Table 1) indicated the main effects of *Wolbachia* and antibiotic treatment were both significant in determining *Elizabethkingia* abundance. There was also a significant interaction between the main effects, whereby the relative abundance of *Elizabethkingia* was increased in the *w*Mel line compared to the wt line, but this effect was negated by addition of antibiotics (Table 1 and Fig 3A). *Wolbachia* and antibiotic treatment were also both significant in determining the relative abundance of the unclassified taxa, with an interaction between the main effects (Table 1). In contrast to *Elizabethkingia*, the unclassified taxa were decreased in mean relative abundance in the *w*Mel line, dropping from a mean *Wolbachia-*corrected relative abundance of 58% in the wt line to 42% in the *w*Mel line (Fig 3B). This effect was reversed in the antibiotic-treated *w*Mel line, which had a higher relative abundance of unclassified taxa on average than the antibiotic-treated wt line (Fig 3B). However, it is notable that the increase in the mean relative abundance across the antibiotic-treated *w*Mel group is due to a high percentage of unclassified taxa in a single mosquito out of the 19 mosquitoes sampled (Fig 2).

**Table 1:** The effect of *Wolbachia* infection and antibiotic treatment on the relative abundance (*Wolbachia*-corrected) of specific genera.

Genus	Factors	F value	df	p
*Elizabethkingia*	*Wolbachia*	5.929	1	.017
	Antibiotics	275.392	1	< .0005
	Wolbachia*antibiotics	6.196	1	.015
Unclassified	*Wolbachia*	5.577	1	.021
	Antibiotics	261.887	1	< .0005
	Wolbachia*antibiotics	10.585	1	.002
*Serratia*	*Wolbachia*	50.439	1	< .0005
	Antibiotics	85.278	1	< .0005
	Wolbachia*antibiotics	50.439	1	< .0005
*Chryseobacterium*	*Wolbachia*	4.285	1	042
	Antibiotics	10.019	1	.002
	Wolbachia*antibiotics	3.407	1	.069
*Weeksella*	*Wolbachia*	.949	1	.333
	Antibiotics	.949	1	.333
	Wolbachia*antibiotics	.949	1	.333
*Pseudomonas*	*Wolbachia*	.291	1	.591
	Antibiotics	19.854	1	< .0005
	Wolbachia*antibiotics	.291	1	.591
*Salinibacterium*	*Wolbachia*	.949	1	.333
	Antibiotics	.949	1	.333
	Wolbachia*antibiotics	.949	1	.333
*Staphylococcus*	*Wolbachia*	1.017	1	.317
	Antibiotics	.882	1	.351
	Wolbachia*antibiotics	.882	1	.351
*Leucobacter*	*Wolbachia*	1.008	1	.319
	Antibiotics	1.008	1	.319
	Wolbachia*antibiotics	1.008	1	.319

*Wolbachia* and antibiotic treatment significantly affected the relative abundance of *Serratia*, again with an interaction between the main effects (Table 1). The mean relative abundance of *Serratia* was increased from 0.2% in wt mosquitoes to 1.4% in the *w*Mel line. However, this difference was negated by antibiotic treatment, with no *Serratia* detected above the lower threshold in either of the antibiotic-treated mosquito lines (Fig 3C). The only taxon showing a significant interaction with *Wolbachia* without an interaction between the main effects was *Chryseobacterium*, which was decreased in mean relative abundance in the *w*Mel line compared to the wt line in both antibiotic-treated and untreated groups (Table 1 and Fig 3D).

### Manipulating the microbiome of *w*Mel-infected *Ae*. *aegypti* by antibiotic treatment does not significantly impact blocking of DENV

**Fig 4:**
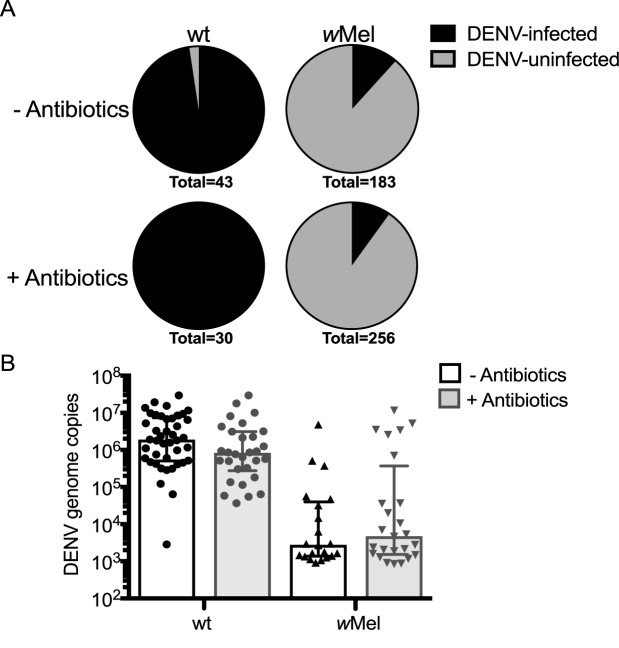
Infection rate and DENV genome copy number are unaffected by manipulation of the microbiome using antibiotic treatment. (A) Percent of infected mosquitoes as determined by qRT-PCR; total numbers of mosquitoes tested per group are listed below each chart. (B) Total number of DENV genome copies per body for all mosquitoes identified as DENV-positive in (A). Statistical analysis was performed using a one-way ANOVA, only the effect of *Wolbachia* was significant. Data shows median and interquartile range.

To ascertain the effect of microbiome manipulation on DENV infection rates, we measured DENV genome copies in individual mosquitoes by qRT-PCR. Infection rates were calculated using the percentage of mosquitoes that returned a DENV-positive qRT-PCR result above our lower detection limit. In the wt line, only one mosquito did not have detectable DENV, with 98% and 100% of non-treated and antibiotic treated mosquitoes, respectively, DENV-positive (Fig 4A). Due to known low DENV infection rates in the *w*Mel line [10,32], a substantially higher number of *w*Mel mosquitoes were blood-fed and tested for DENV-infection than the wt line. While the infection rates of the *w*Mel line were much lower in comparison to the wt line, as expected, the antibiotic-treated *w*Mel and untreated *w*Mel groups had comparable infection rates of 9.8% of 11.5%, respectively (Fig 4A).

To assess whether there was an impact of antibiotic treatment on DENV load in mosquitoes with detectable infection, we compared the number of DENV genome copies in antibiotic-treated and untreated wt and *w*Mel (Fig 4B). There was no effect of antibiotic treatment (F = 0.012, df = 1, p = 0.91) but there was an effect of *Wolbachia* infection (F = 115.9, df = 1, p <0.0001), whereby *w*Mel mosquitoes had significantly lower DENV copies than the wt, as expected [10,36]. Therefore, treatment of *Ae*. *aegypti* with a combination of penicillin-streptomycin-kanamycin did not affect *Wolbachia-*mediated inhibition of DENV load.

## Discussion

In summary, we profiled the microbiome of laboratory-reared *Ae*. *aegypti* to examine the effect of stable infection by *Wolbachia*, and the potential role of the microbiome in *Wolbachia-*mediated DENV blocking. We found that *Wolbachia* has few effects on the microbiome, and that even significant changes to the microbiome caused by our artificial manipulation had no effect on DENV susceptibility in these mosquitoes. This is of particular importance given that *Wolbachia* is likely to encounter diverse microbial environments in the field. Our findings suggest that the microbiome will be largely robust to *Wolbachia* infection and that at least for the species manipulated here, there is no evidence that ‘third parties’ are a critical factor in the expression of *Wolbachia*-mediated DENV blocking.

The microbiome profiling performed in our study identified only a small number of genera present in laboratory-reared *Ae*. *aegypti*, regardless of *Wolbachia* infection status. This low level of microbial diversity is consistent with previous studies of laboratory-reared mosquitoes profiled *via* both culture-dependent and culture-independent methods [23,27,37–39]. While the microbiome composition of mosquitoes is known to differ across environments [31], our detection of *Flavobacteriaceae/Elizabethkingia* and *Enterobacteriaceae* as a substantial component of the microbiome is in keeping with several other studies of laboratory-reared *Aedes* and *Anopheles* species [23,27,29,39,40]. Other genera detected in our study (*Pseudomonas*, *Serratia*, *Chryseobacterium*, *Leucobacter*, *Staphylococcus*, *Weeksella*) have also been reported in previous mosquito microbiome studies [23,38,41,42]. Thus, our 16S rDNA sequencing methods appear robust in their profiling of the *Ae*. *aegypti* microbial community.

Our results indicate that *Elizabethkingia* and the unclassified taxa were the clearly dominant taxonomic groups in both mosquito lines, suggesting that *w*Mel does not require a drastic change in the microbiome composition for stable infection of *Ae*. *aegypti*. This finding is in agreement with a recent study of laboratory-reared *An*. *stephensi*, which also reported *Elizabethkingia* and unclassified *Enterobacteriaceae* as the dominant taxa, and found that infection with *Wolbachia* strain *w*AlbB had no effect [39]. However, it is notable that despite no large shifts in the microbiome in *Wolbachia*-infected *Ae*. *aegypti*, we did detect significant interactions between *Wolbachia* and several taxa: *Elizabethkingia*, *Serratia*, *Chryseobacterium* and the unclassified taxa. Nevertheless, with the exception of *Chryseobacterium*, we were able to alter the nature and/or extent of these interactions by antibiotic treatment, without observing any effects on DENV susceptibility. Similar results were recently observed in *Drosophila*, where *Wolbachia* had significant interactions with the microbiome, but altering the microbiome composition by antibiotic treatment did not change susceptibility to Drosophila C virus [43]. These results suggest that *Wolbachia’s* interactions with the taxa identified in our study are unlikely to contribute to the anti-DENV phenotype in *Ae*. *aegypti*.

To our knowledge, this is the first study to report the effect of antibiotic treatment on *Wolbachia*-mediated DENV blocking in mosquitoes. However, a prior study by Xi and colleagues indicated that treatment of *Wolbachia-*uninfected *Ae*. *aegypti* with antibiotics led to increased DENV titres in the midgut [22], thought to be caused by a down-regulation of immune gene expression in the aseptic mosquitoes [22]. We did not see such a decrease in titres in our wt *Ae*. *aegypti* line following antibiotic treatment, but several differences exist in experimental conditions that may account for our contrasting results, including differing tissues, virus detection/quantification methods, and antibiotic treatment regimes. Although we cannot exclude the possibility that our antibiotic treatment regime may not have targeted taxa that reduce DENV load, there were only three taxa remaining in the wt *Ae*. *aegypti* following antibiotic treatment: *Elizabethkingia* (detected in 19/19 mosquitoes), *Chryseobacterium* (detected in 11/19 mosquitoes), and *Staphylococcus* (detected in 2/19 mosquitoes). Since *Elizabethkingia* and *Chryseobacterium* underwent a significant increase in relative abundance following antibiotic treatment without a corresponding effect on DENV load, it would appear unlikely that the remaining taxa have a strong interaction with DENV. It is notable that there are also differences between our study and that by Xi and colleagues in the DENV genotype (New Guinea C strain [22] versus ET300 strain in the current study) and the mosquito genotype (established *Ae*. *aegypti* Rockefeller/UGAL strain [22] versus generation F5 in the laboratory collected from Babinda, Australia in the current study), which may indicate a role of genotype-by-genotype interactions/effects.

There are several potential limitations of our study. *First*, we used a qRT-PCR approach to quantify virus. While plaque assays would be more informative with respect to quantifying live virus, there is evidence that the two correlate directly by a factor of 100–1000 fold [44]. *Second*, despite shifting the microbiome composition we cannot completely rule out the potential interactions between blocking and any taxa that remain. Lastly, as with all laboratory microbiome studies it is not clear whether these effects will translate to the field. Field populations may have different resident microbiome species or abundances given interactions in field relevant environmental conditions [23,31]. For example, *Elizabethkingia* is commonly found to be a dominant taxon in laboratory-reared mosquitoes (as found in the present study), but is present at much lower relative abundance or absent in field-collected mosquitoes [37,39,45]. As such, future studies should investigate the impact of the microbiome of diverse field mosquitoes on *Wolbachia-*mediated DENV blocking. Nonetheless, our data suggest that the fundamental basis for the anti-DENV phenotype in *w*Mel-infected *Ae*. *aegypti* is unlikely to be caused through specific effects on and/or requiring other microbiota.

In conclusion, we found that stable infection of laboratory-reared *Ae*. *aegypti* with *Wolbachia* strain *w*Mel does not alter the strong dominance of *Elizabethkingia* and unclassified *Enterobacteriaceae* in relation to other genera comprising the microbiome. Importantly, antibiotic treatment did not affect DENV blocking by *w*Mel, despite a measurable alteration in the microbiome composition. Thus, we conclude that *Wolbachia*-mediated DENV blocking does not appear to rely on a specific microbiome composition. These findings fit with recent data from a model system of Semliki Forest virus infection of *Drosophila melanogaster* cells, which indicate that *Wolbachia* inhibits very early stages of the viral replication cycle, and is thus likely to involve an intrinsic mechanism that occurs on a cellular level [46]. Nonetheless, there may be value to profiling the microbiome of wild caught mosquitoes in field populations pre and post *Wolbachia* releases.

## Supporting information

S1 Fig *Wolbachia* levels in mosquitoes subjected to 16s profiling. *Wolbachia* levels in mosquitoes as determined by qPCR after normalisation to *Ae*. *aegypti* house-keeping gene *rps17*. Statistical analysis by unpaired, unequal variance t-test.
(EPS)

S1 Table Relative abundance of genera. (XLSX)

S2 Table Relative abundance of genera, corrected for *Wolbachia*. (XLSX)
